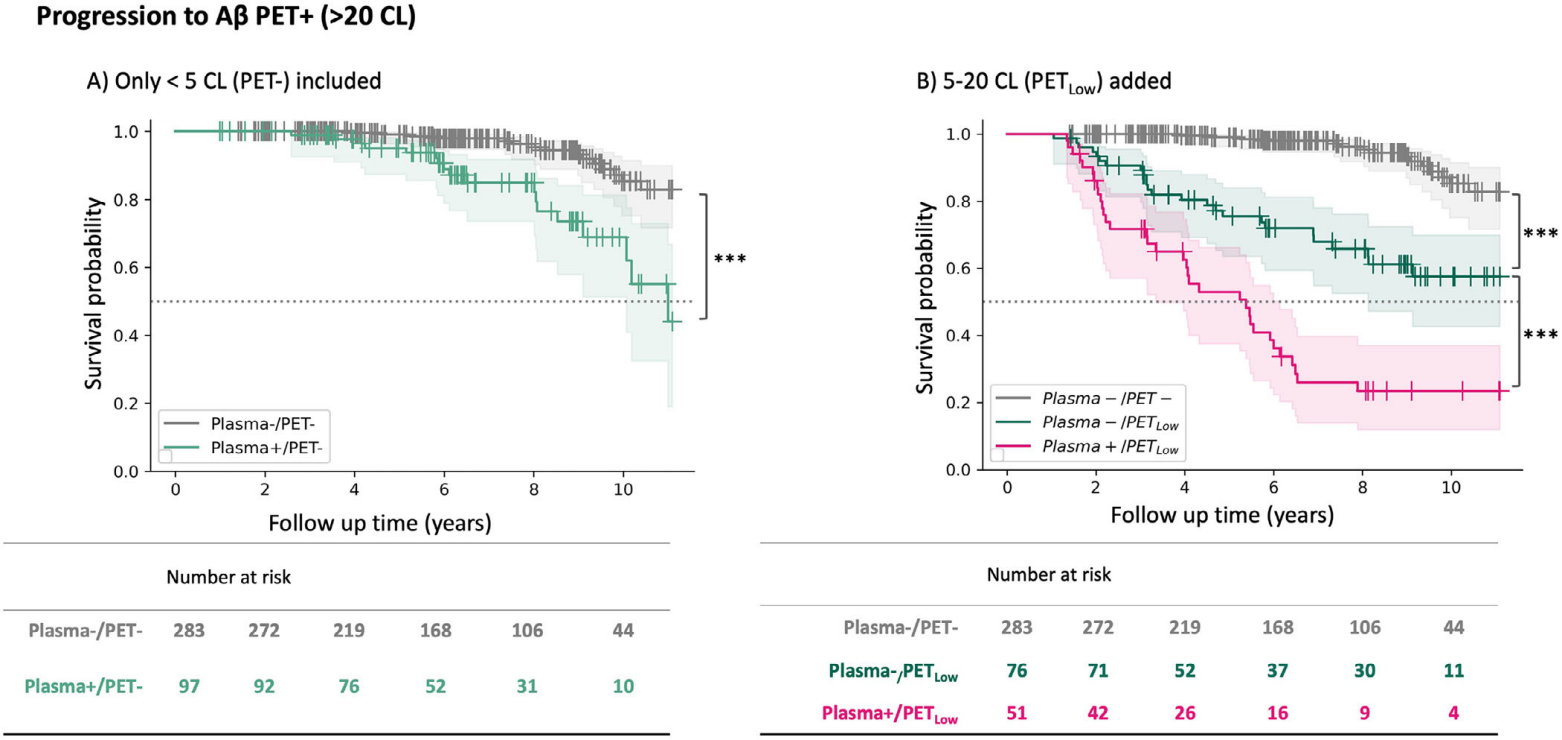# Interpreting positive plasma Aβ42/40 results when amyloid PET is negative

**DOI:** 10.1002/alz70856_105405

**Published:** 2026-01-08

**Authors:** Azadeh Feizpour, Pierrick Bourgeat, Vincent Dore, James D. Doecke, Rodrigo Canovas, Simon M. Laws, Tenielle Porter, Kun Huang, Christopher J Fowler, Michael W Weiner, John C. Morris, Tammie L.S. Benzinger, Suzanne E. Schindler, Randall J. Bateman, Larry Ward, Jurgen Fripp, Colin L. Masters, Victor L. Villemagne, Christopher C. Rowe

**Affiliations:** ^1^ The Florey Institute of Neuroscience and Mental Health, Parkville, VIC, Australia; ^2^ Austin Health, Melbourne, VIC, Australia; ^3^ The Australian e‐Health Research Centre, CSIRO, Brisbane, QLD, Australia; ^4^ Australian E‐Health Research Centre, CSIRO, Melbourne, VIC, Australia; ^5^ Curtin Medical School, Curtin University, Bentley, Western Australia, Australia; ^6^ Collaborative Genomics and Translation Group, Edith Cowan University, Joondalup, Western Australia, Australia; ^7^ Centre for Precision Health, Edith Cowan University, Joondalup, Western Australia, Australia; ^8^ Collaborative Genomics and Translation Group, School of Medical and Health Sciences, Edith Cowan University, Joondalup, Western Australia, Australia; ^9^ Department of Molecular Imaging & Therapy, Austin Health, Melbourne, VIC, Australia; ^10^ The Florey Institute of Neuroscience and Mental Health, Melbourne, VIC, Australia; ^11^ University of California San Francisco (UCSF), San Francisco, CA, USA; ^12^ Knight Alzheimer Disease Research Center, Washington University School of Medicine, St. Louis, MO, USA; ^13^ Washington University in St. Louis, St. Louis, MO, USA; ^14^ The Tracy Family SILQ Center, St. Louis, MO, USA; ^15^ University of Pittsburgh, Pittsburgh, PA, USA

## Abstract

**Background:**

The agreement between plasma Aβ42/40 and Aβ‐PET is approximately 75%, with a large portion of discrepancies due to positive plasma with negative PET results. Questions remain about whether these reflect brain Aβ changes detectable in plasma before PET‐detectable. We aimed to examine these cases over 11 years to assess the risk and timing of progression to Aβ‐PET positivity.

**Method:**

Cognitively unimpaired participants from large‐scale longitudinal studies of AIBL, OASIS, and ADNI underwent baseline Aβ‐PET and plasma Aβ42/40 analysis by IPMS, followed by 1‐7 additional PET scans every 1.5‐3 years. Aβ‐PET was quantified to Centiloid (CL) using the SPM pipeline. Individuals with baseline Aβ‐PET < 20 CL (*n* = 507) were included, with those < 5 CL classified as PET‐, and 5‐20 CL as PET_Low_. Plasma ‐/+ was based on the Aβ42/40 Youden's Index threshold (0.119) corresponding to Aβ‐PET status. We used Kaplan‐Meier method and Cox proportional hazards analysis to assess the risk of progression to PET+ (> 20 CL).

**Result:**

Plasma+/PET‐ (< 5 CL) individuals were at higher risk than Plasma‐/PET‐ of progressing to PET+ (hazard ratio (HR): 3.90 [95% CI: 2.00‐7.61], *p* <0.001), even after matching the groups’ baseline CL values (HR: 3.43 [1.43‐8.26], *p* = 0.010), or adjusting for age, sex, *APOE* ε4 and baseline CL (HR: 2.48 [1.22 ‐ 5.07], *p* = 0.013) (Figure 1A). Plasma+/PET‐ accumulated brain Aβ ∼8 times faster than Plasma‐/PET‐ (1.14 CL/year vs. 0.15 CL/year respectively, *p* <0.001). Plasma+/PET‐ progressors became PET+, on average, 2 years earlier than Plasma‐/PET‐ progressors. Plasma+/PET_Low_ group had faster decline in survival probability than Plasma‐/PET_Low_ (HR: 20.82 [11.28 – 38.42], *p* <0.001 vs. HR: 6.67 [3.51 – 12.65], *p* <0.001) (Figure 1B) but this was driven by higher CL in the Plasma+ group.

**Conclusion:**

Cognitively unimpaired individuals with abnormal plasma Aβ42/40 but negative Aβ‐PET face a significantly increased risk of future positive Aβ‐PET. This provides supporting evidence that brain Aβ pathology can be detected in plasma with IPMS before it is PET‐detectable. Whether this also applies to plasma Aβ42/40 immunoassays warrants investigation.